# THE APPLICATION OF CELL-FREE FETAL DNA (cff-DNA) AND SIBLINGS DNA METHODS IN THE PROCESS OF PATERNITY TEST THROUGH CODIS STR LOCI (CSF1PO, THO1, TPOX, AND vWA)

**DOI:** 10.21010/Ajid.v16i1.2

**Published:** 2021-12-21

**Authors:** Ahmad Yudianto, Arofi Kurniawan, Toetik Koesbardiati, Achmad Faisol, Fery Setiawan, Abdul Hadi Furqoni, Yessi Andriani Fauziah

**Affiliations:** 1 Magister of Forensics, Postgraduate School, Universitas Airlangga, Surabaya-Indonesia; 2 Forensics and Medicolegal Department, Faculty of Medicine, Universitas Airlangga, Surabaya-Indonesia; 3 Forensic Odontology Department, Faculty of Dentistry, Universitas Airlangga, Surabaya-Indonesia; 4 Anthropology Department, Faculty of Social and Political Sciences, Universitas Airlangga, Surabaya- Indonesia; 5 Human Genetics Study Group, Institute of Tropical Diseases, Universitas Airlangga, Surabaya-Indonesia; 6 Doctoral Student, Faculty of Medicine, Universitas Airlangga, Surabaya-Indonesia

**Keywords:** Siblings, cell-free fetal-DNA (cff-DNA), paternity test, pregnant women

## Abstract

**Background:**

The non-invasive cff-DNA and siblings DNA methods are the latest breakthroughs in the forensic identification process. The use of cff-DNA and siblings DNA as non-invasive techniques in the forensic identification process has, hitherto, not been widely proven.

**Methods and Materials:**

This was an analytic observational study. The sample of this study consisted of peripheral blood of women in the second trimester of pregnancy and their two biological children. The kinship analysis was carried out through siblings’ DNA and cff-DNA from the mothers through CODIS STR loci (CSF1PO, THO1, TPOX, and vWA).

**Results:**

The means of allele sharing between full siblings in loci CSF1PO, THO1, TPOX, and vWA were 0 (13.75%), 1 (44.75%), and 2 (41.50%). The allele sharing found in the study is in line with the one in previous research conducted by Wenk (1998) and the theory proposed by O’Connor (2011), indicating that one allele sharing dominates, contrasting with the finding of previous research conducted by Sosiawan (2020) revealing that 2-allele sharing was more superior. The variation is caused by the ethnicity having a different genetic contribution among the population. The variation can be attributed to historical and demographical processes leading to genetic drift.

**Conclusion:**

The mean of SI in 1 allele sharing in CODIS STR loci (CSF1PO, THO1, TPOX, and vWA) has the highest value of 44.5%. The use of cff-DNA of pregnant women as one of the non-invasive techniques can serve as an alternative material in a paternity test.

## Introduction

DNA identification can be used to determine a biological relationship among individuals within a family by comparing the DNA patterns of the individuals (Sukriani, 2012; Yudianto, 2015). As a part of DNA identification, a paternity test is conducted by analyzing the DNA patterns in STR (short tandem repeat) markers. STR is DNA loci composed of the repetition of 2-6 bases. The human genome may contain various numbers and types of base repetition. DNA identification through STR markers is one of the DNA test procedures with high sensitivity because of its high variation rate between loci or individuals (Butler, 2006; Krenke et al., 2005; Hares, 2015).

The role of molecular forensic experts has been widely known since the use of DNA as examination materials. However, DNA as an examination material of molecular forensics is problematic (Butler, 2015). Several issues could complicate the work of a molecular forensic expert during the analysis of DNA samples or in determining the results of DNA examination. Forensic personal identification is often limited by the unavailability of information from parents or children that can be used as comparisons in forensic DNA examination (Butler, et al., 2012; Butler, 2015).

The principles of forensic DNA examination are based on the comparison between alleles of the victims or perpetrators and the alleles originating from their family line (kinship analysis), such as in unborn child disputed cases, paternity disputed cases, and, even, in the identification of mass disaster victims, or war victims. In these situations, the comparison originating from close families such as younger or older siblings is required if the comparison from the parental or filial lines is not available. An identification process using full siblings as a comparison may face the possibility of a mismatched profile of the used DNA loci. Furthermore, the diversity of ethnicities in Indonesia has created the chance of mismatches” in personal identification using siblings DNA profiling (Reid, et al. 2008; Morna, et al. 2009; Lu, et al. 2012; Leclair et al., 2012).

In sexual crime cases causing pregnancy or out-of-wedlock pregnancy cases, a specific technique or method is required to identify who the ’father’ is. Cell-free fetal DNA (cff-DNA) is a non-invasive method in forensic identification processes.

A study conducted by Lo et al. (1998) generates a theory that, during pregnancy, the traffic of molecules naturally occurs. The molecules and cells keep moving from the fetus to the maternal body and vice versa. The studies on the bidirectional traffic relatively have developed rapidly. One of the topics with rapid development is the cff-DNA or the cell-free fetal DNA.

The identification methods (i.e., the non-invasive cff-DNA method and the siblings DNA method) are the breakthroughs in the forensic identification processes. The use of cff-DNA and siblings DNA as non-invasive techniques in forensic identification has not been widely proven. Accordingly, the authors are interested in the study aiming to develop a new alternative of identification materials specifically in forensic DNA profiling to be used in forensic DNA laboratories in Indonesia.

## Materials and Methods

### Research Sample

This study was analytical observational research. The sample consisted of peripheral blood of women in their second trimester of pregnancy and their two biological children. The respondents voluntarily participated in this study as indicated by signing both the consent form and the ethical clearance form. The Faculty of Dentistry issued the ethical clearance through Ethical Clearance Number 329/HRECC.FODM/VI/2021. The number of respondents was 20 families, each of which consisted of a pregnant mother with two biological children. The total number of samples was 60. The study was conducted at Human Genetic Laboratory, Institute of Tropical Diseases [ITD] of Universitas Airlangga.

### Sample Treatment

The first step of this study was the preparation of DNA templates for PCR examination. The DNA templates originated from the peripheral blood of pregnant women and their two children. The mother’s peripheral blood was centrifuged at 1,600 rpm for 10 minutes, and the supernatant was transferred into a centrifuge Eppendorf tube. The DNA from the supernatant inside the tube and the DNA from the pellet in other tubes were isolated by adding DNAzol. 1 ml DNAzol reagent was added into 0.1 ml of the child’s peripheral blood. All of the samples were incubated at room temperature for 5 minutes. Next, the samples were centrifuged at 10,000 rpm for 10 minutes at 4°C. The viscous supernatant was transferred into new tubes. 0.5 ml of 100% ethanol was added to the new tubes. The samples were incubated at room temperature for 1-3 minutes and centrifuged at 4,000 rpm at 4 °C for 2 minutes. The supernatant was later removed. The pellets were cleansed twice with 0.8-1 ml of 75% ethanol. The pellets containing DNA were diluted in 25-30 µl of distilled water and stored at -20 °C (Chomcszynski P, et al. 1997: Fung WK, et al.2004).

### PCR Amplification

The DNA amplification process was conducted through Polymerase Chain Reaction process [PCR] (PowerPlex® 21Systems, Promega, USA) targeting specific DNA sequences to replicate the isolated DNA samples. All 60 samples were amplified using 4 primers of Combined DNA Index System Short Tandem Repeats [CODIS-STRs], namely: **CSF1PO** [5’-AACCTGAGTCTGCCAAGGACTAGC-3’ and 5’-TTCCACACACCACTGGCCATCTTC-3’], **THO1** [5’-CTGGGCACGTGAGGGCAGCGTCT-3’and 5’-TGCCGGAAGTCCATCCTCACAGTC-3’], **TPOX** [5’-ACTGGCACAGAACAGGCATCTAGG-3’ and 5’-GGAGGAACTGGGAACCACACAGGT-3’]. and **vWA** [5’-CCTAGTGGATGATAAGAATAATCAGTATG-3 and 5’- GGACAGATGATAAATACATAGGATGGATGG-3’].

The PCR reaction used 10 μl of the DNA templates, 12.5 μl of PCR Mastermix, and reverse and forward primers @ 2.5 μl. Nuclease Free Water was added into the PCR tubes until reaching the volume of 25 μl. The amplification of PCR loci THO1, TPOX, and vWA was conducted as follows: Initial denaturation at 96°C-2 minutes, 10 cycles [Subsequent denaturation at 94°C-1 minute, Annealing at 64°C-1 minute, Extension at 70°C - 1 minute 30 seconds], 30 cycles [Denaturation at 90°C-1 minute, Annealing at 64°C-1 minute, and Extension at 70°C-1 minute 30 seconds]. The amplification of PCR locus CSF1PO was done as follows: at 96°C-2 minutes, next [at 94°C-1 minute, at 64°C-1 minute, at 70°C -1,5 minutes, for 10 cycles], and, then, [at 90°C-1 minute, at 64°C-1 minute, at 70°C-1,5 minutes, for 30 cycles] (Promega Corp 2001).

### Gel Electrophoresis

**Figure 1:**
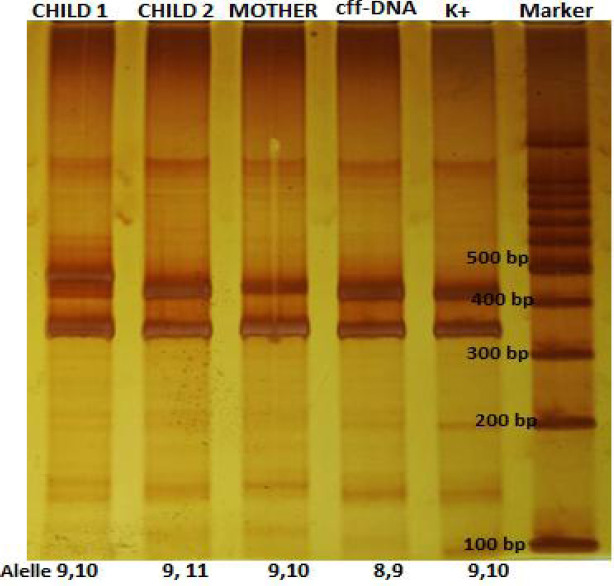
PCR visualization of locus CSF1PO [321bp – 357bp], M [marker 100bp], Child 1, Child 2, Mother, cff-DNA and K+ [K562].

The results of PCR examination were visualized using vertical electrophoresis with 6% polyacrylamide agarose gel electrophorese [PAGE] [Bio-Rad Mini-PROTEAN®] with silver nitrate staining [Figure 1].

### Allele Samples

The results of PCR visualization reading were in the forms of alleles of each locus with K562 control [Table 1]. The alleles were matched in each family consisting of [a (pregnant) mother, cff-DNA from the fetus, and both children] and the allele frequency rate [Table 2]. The analysis was based on the frequency of allele sharing using sibling analysis [alleles shared between the two siblings and the fetus’ cff-DNA] of each locus of CODIS-STRs [CSF1PO, THOI, TPOX and vWA] by referring to the use of the shared alleles [Table 3].

**Table 1:** Allele profile of CODIS-STRs of the samples

CODIS STR LOCI									

Family	Code	CSF1PO		THOI		TPOX		vWA	
**1**	**Child 1**	9	11	9	9	8	9	15	17
	**Child 2**	9	10	9	10	8	9	16	17
	**Mother**	9	10	9	9	8	9	15	17
	**cff-DNA**	8	9	9	9.3	9	10	17	17
**2**	**Child 1**	9	10	9	9	8	9	15	16
	**Child 2**	8	9	9	9.3	8	10	15	16
	**Mother**	7	9	8	9	7	8	15	17
	**cff-DNA**	9	10	9	10	8	9	16	17
**3**	**Child 1**	9	10	8	9	8	9	15	17
	**Child 2**	9	9	7	9	8	9	15	17
	**Mother**	8	9	7	8	8	9	16	17
	**cff-DNA**	9	10	8	10	8	9	16	17
**4**	**Child 1**	8	9	9	10	8	9	13	15
	**Child 2**	7	8	7	8	8	9	14	15
	**Mother**	7	8	8	9	8	9	13	14
	**cff-DNA**	8	9	9	9.3	9	10	14	15
**5**	**Child 1**	9	10	9	9	8	9	15	17
	**Child 2**	8	9	9	9.3	8	9	17	17
	**Mother**	8	10	9	9.3	7	9	15	17
	**cff-DNA**	9	10	9	9	8	9	15	17
**6**	**Child 1**	9	10	9	9	8	9	15	17
	**Child 2**	8	9	9	9.3	9	10	17	17
	**Mother**	8	9	9	9	8	9	15	17
	**cff-DNA**	8	9	9	9.3	9	10	17	17
**7**	**Child 1**	9	11	9	9.3	8	9	15	17
	**Child 2**	9	10	9.3	10	8	9	16	17
	**Mother**	9	11	9.3	9.3	8	9	15	17
	**cff-DNA**	8	9	9	9.3	9	10	17	17
**8**	**Child 1**	9	11	9	9	8	9	15	17
	**Child 2**	9	10	8	10	8	9	16	17
	**Mother**	9	10	8	9	7	8	16	17
	**cff-DNA**	9	10	9	10	8	9	16	17
**9**	**Child 1**	9	11	9	9	8	9	15	17
	**Child 2**	9	10	9	10	8	9	16	17
	**Mother**	9	11	9	9	8	9	15	17
	**cff-DNA**	9	10	9	10	8	9	16	17
**10**	**Child 1**	9	10	9	9	8	9	15	17
	**Child 2**	8	11	9	9	8	9	15	17
	**Mother**	8	10	9	10	8	9	16	17
	**cff-DNA**	9	10	9	9	8	9	15	17
**11**	**Child 1**	9	10	9	10	8	9	16	17
	**Child 2**	9	10	9	9	8	9	15	17
	**Mother**	9	11	9	9	8	9	15	17
	**cff-DNA**	9	10	9	10	8	9	16	17
**12**	**Child 1**	9	11	9	9	8	9	15	17
	**Child 2**	9	10	9	10	8	9	16	17
	**Mother**	9	10	9	9	8	9	15	17
	**cff-DNA**	9	11	9	9	8	9	15	17
**13**	**Child 1**	9	10	9	10	8	9	16	17
	**Child 2**	9	11	9	9	8	9	15	17
	**Mother**	9	10	9	10	8	9	16	17
	**cff-DNA**	9	10	9	9	8	9	15	17
**14**	**Child 1**	8	11	9	9	8	9	16	17
	**Child 2**	8	10	9	10	8	9	16	17
	**Mother**	7	8	9	9	8	9	14	16
	**cff-DNA**	8	10	9	10	8	9	16	17
**15**	**Child 1**	9	10	9	9	8	9	15	17
	**Child 2**	8	11	9	9	8	9	14	17
	**Mother**	8	9	9	10	8	9	14	16
	**cff-DNA**	9	11	9	9	8	9	15	16
**16**	**Child 1**	9	10	8	10	8	9	16	17
	**Child 2**	9	10	8	9	8	9	15	17
	**Mother**	8	10	7	8	8	9	15	17
	**cff-DNA**	9	10	8	10	8	9	16	17
**17**	**Child 1**	9	11	9	9	8	9	15	17
	**Child 2**	9	10	9	10	8	9	15	17
	**Mother**	9	10	8	9	8	9	14	15
	**cff-DNA**	9	11	9	9	8	9	15	17
**18**	**Child 1**	9	10	9	10	8	9	16	17
	**Child 2**	9	11	9	9	8	9	15	17
	**Mother**	7	9	9	10	8	9	16	17
	**cff-DNA**	9	10	9	9	8	9	15	17
**19**	**Child 1**	7	8	9	9	8	9	15	17
	**Child 2**	6	7	9	10	8	9	16	17
	**Mother**	6	7	8	9	8	9	16	17
	**cff-DNA**	6	8	9	10	8	9	16	17
**20**	**Child 1**	9	10	9	9	8	9	15	17
	**Child 2**	8	10	8	9	8	9	15	17
	**Mother**	8	9	8	10	8	9	16	17
	**cff-DNA**	9	10	9	10	8	9	16	17

**Table 2:** Allele frequency in the samples of CODIS STR LOCI CSF1PO, THO1, TPOX, vWA [n: 160].

Loci	Allele	Frequency	Loci	Allele	Frequency
**CSF1PO**	6	0.01875	**THOI**	7	0.02500
	7	0.05000		8	0.10000
	8	0.11875		9	0.61250
	9	0.40625		9.3	0.07500
	10	0.28125		10	0.18750
	11	0.12500			
**vWA**	13	0.01250	**TPOX**	7	0.01875
	14	0.04375		8	0.47500
	15	0.25625		9	0.46875
	16	0.21875		10	0.03750
	17	0.46875			

**Table 3:** Percentage of Sibship Index [**SI**] in the research

Probability Ratio in Sibship Indices (**SI**)	Strength	Percentage in the research
< 1	Weak	10%
1 - 10	Moderate	10%
10 -100	Strong	20%
>100	Very Strong	55%

## Results

The mean DNA level within the peripheral blood sample was **696 ± 4.23** ng/μl with a purity range of 1.02-1.76. Meanwhile, the mean DNA level of the cff-DNA was **36 ± 5.30** ng/μl with a purity range of 1.05-1.67. The DNA levels of the samples were acceptable in the DNA profiling, specifically in the paternity test. The test required approximately **20** μg/ml of DNA for typing (Yudianto, 2015). The process continued with PCR amplification through 4 primers of CODIS-STRs loci (CSF1PO, THO1, TPOX, and vWA) and the visualization of PCR results through PAGE with silver nitrate staining. The result of PCR visualization is presented in the following Figure 1.

Table 1 shows the allele profile of CODIS-STR of each sample derived from 20 samples consisting of child-1, child-2, mother, and cff-DNA.

Table 2 shows the allele frequency in the samples of CODIS STR LOCI using CSF1PO, THO1, TPOX, vWA from the 160-total number of samples. It shows the frequency of each locus that consists of CSF1PO, vWA, THO1, and TPOX.

**Figure 2:**
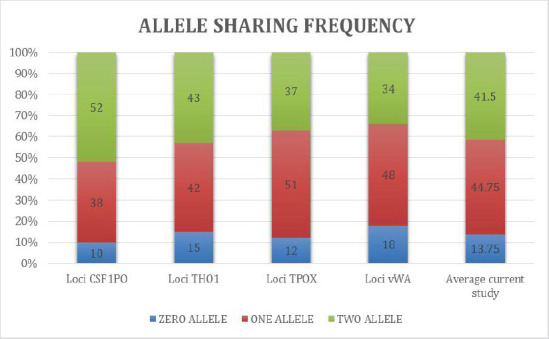
The allele sharing frequency in the samples of CODIS STR LOCI CSF1PO, THO1, TPOX, vWA [n : 160]

Figure 2 shows the allele sharing frequency from each of sample using CODIS STR locus CSF1PO, THO1, TPOX, and vWA from the total number of samples are 160 samples.

The result of the Sibship Index [**SI**] examination is presented in Table 3 below.

Table 3 shows the Sibship Indices (SI), illustrating that 75% of the siblings, including the cff-DNA of each person has very strong SI exceeding 100, which is 55% (very strong), and 20% has a sibship ratio of 10-100 (strong).

## Discussion

The process of paternity test makes use of parents as comparisons so that the obtained statistical result is very close to 100% or 99.99% (Untoro et al. 2009). The unavailability of information from the parents or children that can be used as comparisons in forensic DNA examinations hinders the forensic DNA analysis (Omran, et al. 2009; O’Connor, 2011). Unlike DNA examination with parental DNA as the comparison, the use of siblings DNA in personal identification may not have 100% precision. The siblings who are used as the comparison can be born children or unborn fetuses. In pregnant women at the age of 1-2 trimesters of the pregnancy, the blood circulatory system contains cell-free fetal DNA (cff-DNA), the DNA fragments originating from the fetus penetrating the maternal circulation. These fragments can be identified through the DNA isolation method from maternal plasma samples (Kido, 2003; Venkanna et al., 2008; Sosiawan et al., 2015; Yudianto, et al. 2019). In sibship/sibling analysis, allele sharing plays a significant role. The allele sharing in determining full siblings is useful in connecting the alleles when both are involved. Statistically, full siblings have the precision probability of 2 alleles as much as 25%. This probability value equals not sharing any allele or 0 (zero) shared alleles; meanwhile, the probability of 1 shared allele is 50% (O’Connor, 2011; Maeda et al., 2015; Marano et al., 2019; Sosiawan et al., 2020; Yudianto et al., 2021).

In this study, the means of allele sharing between full siblings in loci CSF1PO, THO1, TPOX, and vWA were 0 allele (13.75%), one allele (44.75%), and 2 alleles (41.50%) (shown by figure 2). The means of allele sharing found in the study was in line with the one in previous study conducted by Wenk (1996) and a theory proposed by O’ Connor (2011) showing that 1 allele sharing dominates namely 44.75%. This finding contrasts with the previous research conducted by Sosiawan (2020), revealing that 2-allele sharing was more superior. The variation was caused by the ethnicity that had a different genetic contribution among the population. The variation could be attributed to historical and demographical processes leading to genetic drift (Maeda, et al., 2005; Venkanna et al., 2008).

The allele sharing is a genetic variation inherited from both parents. All individuals are a part of a population due to marriage between the individuals and share the same gene pool. Gene pool is the collection of all genes/alleles within a population (Mangoendidjojo W, 2014). The result of Sibling Indices (SI) showed that 75% of pairs of full siblings, including cff-DNA, had SI more than 10 (strong and very strong) (shown by Table 3). This finding implies that the application of STR loci CSF1PO, THO1, TPOX, and vWA would be very predictive in identifying full siblings.

The Hardy-Weinberg Equilibrium principle states that the frequency of genes and genotypes tend to be stable through generations in a balanced population. This condition can be found in a large population with random marriages and no intervention regulating specific characteristics (Rong et al., 2012; Sosiawan et al., 2015; Artadana et al., 2018 ).

## Conclusion

A paternity test is based on comparison processes of the alleles originating from the parents. If the alleles from the parents are unavailable, the process can be conducted by comparing the alleles originating from full siblings or cff-DNA of pregnant women. The findings of this study show that the mean of SI in 1 allele sharing has the highest value, as much as 44.5% in CODIS-STR loci (CSF1PO, THO1, TPOX, and Vwa). The application of cff-DNA originating from pregnant women serves as an alternative material in a paternity test, a non-invasive technique.

### Conflict of Interest:

The authors declare that there is no conflict of interest associated with this study.

**List of Abbreviations:**
cff-DNA –: cell-free fetal Deoxyribonucleic Acid
CODIS –: Combined DNA Index System
STR –: Short Tandem Repeat
PCR –: Polymerase Chain Reaction
PAGEs -: polyacrylamide agarose gel electrophoreses
SI -: Sibship Indices
